# Guided Lamb Wave Array Time-Delay-Based MUSIC Algorithm for Impact Imaging †

**DOI:** 10.3390/s24061882

**Published:** 2024-03-15

**Authors:** Fei Zheng, Shenfang Yuan

**Affiliations:** Research Center of Structural Health Monitoring and Prognosis, State Key Laboratory of Mechanics and Control for Aerospace Structures, Nanjing University of Aeronautics and Astronautics, 29 Yudao Street, Nanjing 210016, China; zfd@nuaa.edu.cn

**Keywords:** composite, guided Lamb wave, multiple signal classification, impact monitoring, array time delay

## Abstract

Composite materials, valued in aerospace for their stiffness, strength and lightness, require impact monitoring for structural health, especially against low-velocity impacts. The MUSIC algorithm, known for efficient directional scanning and easy sensor deployment, is gaining prominence in this area. However, in practical engineering applications, the broadband characteristics of impact response signals and the time delay errors in array elements’ signal reception lead to inconsistencies between the steering vector and the actual signal subspace, affecting the precision of the MUSIC impact localization method. Furthermore, the anisotropy of composite materials results in time delay differences between array elements in different directions. If the MUSIC algorithm uses a fixed velocity value, this also introduces time delay errors, further reducing the accuracy of localization. Addressing these challenges, this paper proposes an innovative MUSIC algorithm for impact imaging using a guided Lamb wave array, with an emphasis on time delay management. This approach focuses on the extraction of high-energy, single-frequency components from impact response signals, ensuring accurate time delay measurement across array elements and enhancing noise resistance. It also calculates the average velocity of single-frequency components in varying directions for an initial impact angle estimation. This estimated angle then guides the selection of a specific single-frequency velocity, culminating in precise impact position localization. The experimental evaluation, employing equidistantly spaced array elements to capture impact response signals, assessed the effectiveness of the proposed method in accurately determining array time delays. Furthermore, impact localization tests on reinforced composite structures were conducted, with the results indicating high precision in pinpointing impact locations.

## 1. Introduction

Composite material structures are increasingly used in both civilian and military aviation and aerospace vehicles due to their lightweight, high-strength, high-modulus, and fatigue-resistant properties. These appropriate properties enhance structural efficiency and reduce structural weight [1,2,3]. However, these structures are not without their vulnerabilities, particularly in terms of their sensitivity to impact loads [4,5,6,7].

When subjected to impacts from external objects, composite material structures are prone to internal matrix cracking, fiber breakage, debonding, and delamination, resulting in invisible damages. These damages can severely degrade the structure’s mechanical performance, substantially reducing its load-bearing capacity and posing significant safety risks. Consequently, impact monitoring has become a critical focus in the structural health monitoring (SHM) [8,9,10] of composite materials. Achieving high-precision and real-time monitoring, especially through precise online localization of impacts, is essential.

The use of guided Lamb waves, known for their long-distance propagation in structures and sensitivity to minor damages, is emerging as one of the most promising methods for the online monitoring of composite materials [11,12,13]. Acoustic emission technology specializes in detecting acoustic signals produced by abrupt structural changes. It employs methods such as zoning, hit sequence analysis, and triangulation to pinpoint the event’s location [14]. However, it provides only approximate flaw localization and lacks precise accuracy. For example, using triangulation, it can generally locate a flaw from three sensors but struggles to differentiate between several flaws in the same area. Additionally, the large size of traditional sensors limits their integration into structures. The piezoelectric electromechanical impedance monitoring method [15], flexible eddy current monitoring method [16], comparative vacuum monitoring method [17], and intelligent coating monitoring method [18] can be used to detect structural damage. However, they are unable to monitor structural strain, load, and deformation, thus cannot achieve structural impact monitoring. Guided Lamb wave structural health monitoring technology supports multiparameter monitoring, including impact and damage, with capabilities for damage identification, localization, and quantitative diagnosis. This technology is applicable to a wide range of structures and materials, including metals and composite materials. It offers flexible application methods that do not depend on load conditions and supports both onboard online and ground offline monitoring. Additionally, it enables the integration of a piezoelectric sensor network with aircraft structures for long-lasting and highly reliable integration. For instance, in the field of pipeline damage detection, Xu et al. [19] explore guided Lamb wave technology for pipeline damage detection, developing models for wave propagation in straight and complex pipes and analyzing echo signal impacts. They introduce a wavelet-based denoising method to enhance detection accuracy by minimizing noise.

The MUltiple SIgnal Classification (MUSIC) algorithm is a high-resolution method based on spatial spectrum estimation, designed to perform unbiased estimation using the orthogonality of signal and noise subspaces [20,21]. The MUSIC algorithm, by employing a dense sensor array configuration, not only facilitates deployment within complex composite material structures and confined spaces, but also enables extensive structural direction scanning, covering a broad monitoring range. In comparison to phased array method [22] that can only detect damage, the MUSIC algorithm is capable of both passive impact source localization and active damage localization. Furthermore, when utilizing a one-dimensional linear array, unlike spatial filtering methods [23] that can only assess the direction of damage, the MUSIC algorithm can simultaneously determine the angle and distance of the signal source, demonstrating its significant advantage in precise localization. Moreover, due to its superior performance in resolving closely spaced or overlapping signal sources, the MUSIC algorithm has been widely applied in various fields [24,25,26]. In traditional applications, such as radar signal processing, sonar detection, and seismic wave analysis, the MUSIC algorithm has demonstrated significant effectiveness in locating and identifying signal sources [27,28,29].

In recent years, with the rapid development of SHM, the MUSIC algorithm has been introduced for impact localization. Yuan [30], Engholm [31], and Yang [32] were pivotal in introducing the MUSIC algorithm for impact localization. Their approach utilized guided Lamb wave signals generated from structural impacts to determine the direction of these impacts based on the far-field signal model. When impacts occur near sensor arrays, the MUSIC algorithm faces challenges due to inaccuracies in calculating the array’s steering vector, leading to poor localization. Yuan’s team [33] addressed this by developing the near-field 2D-MUSIC algorithm, significantly enhancing localization accuracy in near-field scenarios. This innovative approach not only improves the handling of impacts close to arrays, but also enables simultaneous localization of the impact’s angle and distance. Zhang et al. [34] explore impact source localization in vibrational conditions, introducing the adaptive-filtering enhanced multiple signal classification (AF-MUSIC) method. This method reduces noise in array data due to vibrations through adaptive filtering. In Zhu’s team’s research [35], they merged the MUSIC algorithm with an Artificial Neural Network (ANN). This merger enabled the MUSIC algorithm to extract critical organized feature matrices from the ANN’s output, leading to more than 90% accuracy in pinpointing impact locations.

However, in the aforementioned MUSIC-like methods, the time delay errors in the signal reception of array elements are overlooked, and the anisotropy of composite materials, which can lead to variations in time delay across array elements in different directions, is also not considered. Impact response signals are broadband, and in practical engineering applications, errors in the time delay of signal reception by array elements can lead to phase errors in the array response signal. This results in the calculated steering vector not being equivalent to the actual signal subspace, causing the array’s steering vector for the impact source position to be non-orthogonal to the noise subspace, thereby reducing the precision of the MUSIC impact localization method. On the other hand, the anisotropy of composite materials leads to noticeable time delay differences among array elements in different directions. If a fixed velocity value is used in the MUSIC algorithm, this also introduces phase errors in the array response signal, further decreasing the accuracy of the MUSIC impact localization method.

For the extraction of single-frequency components from broadband waves, Continuous Wavelet Transform (CWT) techniques have been applied to analyze nonlinear broad-band waves, enabling the isolation of single-frequency components within them. For ex-ample, Xu et al. [36] introduced a sign coherence factor (SCF)-based algorithm leveraging laser-generated Lamb waves for defect detection in plates, enhancing image quality, robustness, and signal clarity through adaptive search and multi-frequency analysis. The Shannon wavelet is designed based on the Shannon sampling theorem, exhibiting exceptional frequency band division capabilities, which makes it outstanding in frequency analysis. The complexity of the Shannon wavelet, through the introduction of its complex form, can provide information about the signal’s phase. Daubechies wavelets, while maintaining a certain level of smoothness, possess good compact support, making them excellent in image compression and signal denoising. The Haar wavelet, with its simple structure, is widely used in image processing for rapid computations. The Morlet wavelet, due to its excellent localization properties, is particularly suited for feature extraction in signal analysis. The complex Shannon wavelet, with its clear frequency band division, performs exceptionally well in applications sensitive to frequency characteristics, such as modulation signal analysis. Therefore, this paper focuses on using the complex Shannon wavelet to extract single-frequency components from impact response signals.

To address the challenges outlined, a guided Lamb wave array time-delay-based MUSIC algorithm for impact imaging is proposed. This method involves extracting high-energy single-frequency components from impact response signals to precisely determine the time delays of each array element while enhancing noise resistance. Additionally, it uses the average velocity of single-frequency components in various directions to initially estimate the impact angle. This estimation is then utilized to select the appropriate single-frequency velocity for the corresponding angle, enabling precise localization of the impact. The efficacy of this approach is validated in composite structures, where equidistantly spaced array elements receiving impact response signals are used to assess the method’s accuracy in determining array time delays. Furthermore, impact localization experiments on reinforced composite structures show that the method achieves high localization precision.

In this study, we introduce an enhanced MUSIC algorithm designed for precise im-pact localization within composite structures, addressing critical challenges in structural health monitoring. This advancement is particularly pivotal across various domains where composite materials play a foundational role. In aerospace, our algorithm promises to elevate the safety and reliability of aircraft by improving the precision of low-velocity impact detection in composite material structures. Similarly, in the realms of wind energy and offshore platforms, it offers a robust solution for monitoring the integrity of critical structures against environmental and operational stresses. The automotive industry, with its growing reliance on composite materials for vehicle manufacturing, stands to benefit from our algorithm through enhanced impact monitoring and damage assessment, con-tributing to greater vehicle safety and durability. Furthermore, in civil engineering applications, such as the monitoring of bridges and skyscrapers, our algorithm facilitates the timely identification and localization of structural damage, thus ensuring safety and pro-longing the service life of these large-scale structures. Through these applications, our im-proved MUSIC algorithm underscores a significant leap forward in the field of structural health monitoring, showcasing its potential to impact a wide range of industries by ensuring the integrity and longevity of composite material structures.

This paper is organized as follows: Section 2 introduces the guided Lamb wave array time-delay-based MUSIC algorithm for impact imaging. In Section 3, the effectiveness of the proposed method in precisely determining array time delays is evaluated. Section 4 details the execution of impact localization experiments on a reinforced composite material structure. Finally, Section 5 gives the conclusion.

## 2. Array Time-Delay-Based MUSIC Algorithm

**Figure 1:**
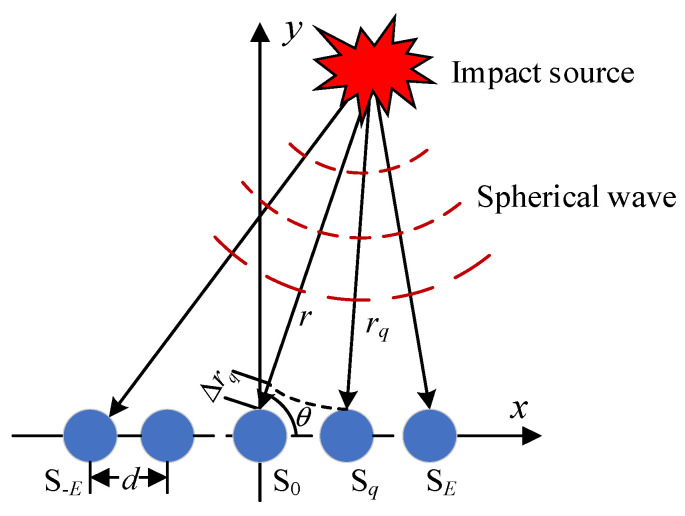
Uniform linear sensor array’s near-field signal model.

During the service life of the composite structure, impacts are monitored using the array time-delay-based MUSIC algorithm, as shown in Figure 1. The sensor array comprises 2*E* + 1 elements, each separated by a distance *d*. The definition of ‘*E*’ refers to the sequence number of array elements. The central sensor, labeled S_0_, serves as the reference element. In near-field conditions, the array captures signal responses in the form of spherical wavefronts. The distance and angle to the signal source differ for each sensor, with angles being measured relative to the *x*-axis. When an impact occurs, the sensors in array S capture the waveforms.

To precisely identify time delays across array elements and improve noise resistance, the Shannon wavelet technique is used to extract a specific frequency from the wide-ranging frequency components of impact response signals.

Signal extraction at a specific frequency is achieved through wavelet-based filtering. Utilizing the complex Shannon wavelet, derived from the sinc function, ensures excellent frequency localization due to its minimal time decay. This makes it exceptionally suitable as a passband filter, especially for extracting signals with narrow bandwidths [37,38,39]. The definition of the complex Shannon wavelet function is as follows:

(1) $$\phi \left(t\right)={\left(f_{k}\right)}^{1/2}\mathrm{sinc}\left(f_{k}t\right)e^{2i\pi f_{z}t},$$

where *f_z_* corresponds to the central frequency of the Shannon mother wavelet, while *f_k_* indicates its bandwidth range. The definition of the sinc function is as follows:

(2) $$\mathrm{sinc}\left(t\right)=\{\begin{array}{cc} 1, & t=0\\ {\left(\pi t\right)}^{-1}\cdot \sin\left(\pi t\right), & t\neq 0 \end{array},$$

The complex Shannon wavelet undergoes a Fourier transform, expressed as:

(3) $$\Psi \left(f\right)=\{\begin{array}{cc} {\left(f_{k}\right)}^{-1/2}, & f_{z}-0.5f_{k}\leq f<f_{z}+0.5f_{k}\\ 0, & \mathrm{others} \end{array},$$

where the term “others” in the formula refers to all frequencies that do not fall within the specified interval.

In the frequency domain, the Shannon wavelet appears as a symmetrical rectangular window centered on the frequency *f_z_*. It features a uniform amplitude within its passband and zero amplitude outside it. The Shannon wavelet’s frequency passband extends from $\left[f_{z}-0.5f_{k},f_{z}+0.5f_{k}\right]$.

The complex Shannon wavelet transform, adept at isolating ultra-narrow-bandwidth signals, enables the treatment of the extracted signal as a single frequency component. The application of this transform to a signal *s*(*t*) is detailed in Equation (4).

(4) $$T_{x}\left(s_{c},t_{f}\right)={\left(s_{c}\right)}^{-1/2}\int_{R}s\left(t\right){\left[{\left(f_{k}\right)}^{1/2}\sin c\left({\left(s_{c}\right)}^{-1}\cdot (t-t_{f})\cdot f_{k}\right)e^{{\left(s_{c}\right)}^{-1}\cdot (t-t_{f})\cdot 2i\pi f_{z}}\right]}^{*}dt,$$

where *s_c_* and *t_f_* are the scale and time factors, respectively, with * denoting conjugation. The coefficient of the wavelet transform of *s*(*t*) at scale *s_c_* represents the bandwidth component centered around *f_k_*/*s_c_*. By maintaining fixed *f_k_* and *f_z_* in the Shannon mother wavelet and adjusting *s_c_*, a highly specific narrow-frequency component can be extracted from the signal. To isolate a particular frequency component *F_z_*, the scale factor *s_c_* is determined based on the correlation between the wavelet’s frequency and the scale factor, as depicted in Equation (5):

(5) $$s_{c}=f_{z}f_{s}/F_{z},$$

where *f_s_* denotes the sampling frequency.

To localize impacts, the noise subspace is derived by determining the covariance matrix of the single-frequency signal. $U_{N}$ represents the noise subspace, which is constituted by the eigenvectors associated with the smaller eigenvalues in the signal’s spectrum.

The eigenvector corresponding to the maximum eigenvalue of the covariance matrix of array signals indicates the propagation direction of the array signal. The eigenvalues of the covariance matrix represent the magnitude of the array signal’s projection along the respective eigenvector. Therefore, it is essential to compare the eigenvalues of the extracted impact response signals at different frequencies. A higher amplitude at a particular frequency results in a larger corresponding eigenvalue. Consequently, selecting single-frequency signals with higher energy is necessary to enhance the differentiation between large and small eigenvalues.

In this method, S_0_ functions as the reference sensor. The signal it receives is represented as

(6) $$s(t)=u(t)e^{j(\omega_{0}t-\frac{\omega_{0}}{v}r)},$$

where *u*(*t*) symbolizes the amplitude of the signal, while *r* denotes the distance between the impact source and the reference piezoelectric lead zirconate titanate (PZT) sensor S_0_. *v* is the velocity of the guided Lamb wave component with frequency *ω*_0_.

Given the frequent presence of background noise, the array signal observed in near-field conditions is expressed as

(7) X(t)=A(r,θ)S(t)+N(t),

where N(t) defined as $N\left(t\right)={\left[n_{-E}\left(t\right),n_{-E+1}\left(t\right),\ldots ,n_{E}\left(t\right)\right]}^{T}$ represents the background noise, while X(t) is denoted as ${\left[x_{-E}\left(t\right),x_{-E+1}\left(t\right),\ldots ,x_{E}\left(t\right)\right]}^{T}$. The array’s steering vector at position (r,θ) is established as per Equation (8).

(8) $$A\left(r,\theta \right)={\left[a_{-E}\left(r,\theta \right),a_{-E+1}\left(r,\theta \right),\ldots ,a_{E}\left(r,\theta \right)\right]}^{T},$$

where $a_{q}\left(r,\theta \right)$ denotes the phase delays arising from positional changes of the sensors.

(9) $$a_{q}\left(r,\theta \right)=e^{-j\omega_{0}\tau_{q}},\;q=-E,\ldots ,E,$$

in which the time delay is evaluated as follows,

(10) $$\tau_{q}=\frac{\Delta r_{q}}{v}=\frac{r-r_{q}}{v}=\frac{r-\sqrt{r^{2}+q^{2}d^{2}-2rqd\cos\theta }}{v},$$

where $r_{q}$ represents the distance between the *q*th sensor and the position at (r,θ), while $\tau_{q}$ denotes the differential arrival time at each sensor relative to the reference sensor.

Ultimately, the calculation of the spatial spectrum is performed as follows,

(11) $$P_{MUSIC}\left(r,\theta \right)=\frac{1}{A^{H}\left(r,\theta \right)U_{N}U_{N}^{H}A\left(r,\theta \right)},$$

where “H” represents the operation of conjugate transposition of a matrix.

The preceding analysis highlights that in the MUSIC algorithm, time delay is a key factor for calculating the steering vector. This makes the algorithm’s ability to localize impacts highly reliant on the precise acquisition of time delays. Accurate determination of these time delays in signals received by each array element is therefore vital for achieving precise impact localization.

## 3. Validation of Precise Acquisition of Array Time Delays

This section first verifies the effectiveness of the proposed method in extracting single-frequency signals. Subsequently, it evaluates the effectiveness of the proposed method in precisely determining array time delays. Additionally, it compares this approach with other time delay acquisition methods, namely, fixed threshold and adaptive threshold techniques.

### 3.1. Verification of Single-Frequency Signal Extraction

**Figure 2:**
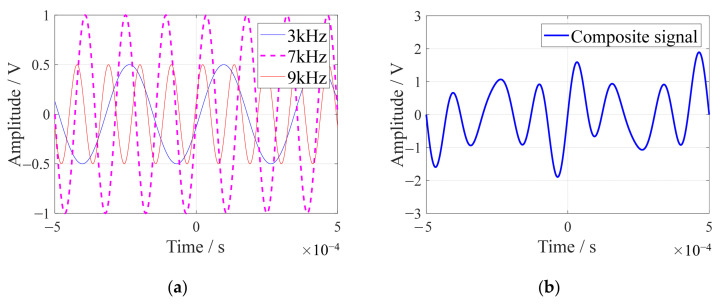
Sine waves and their composite signal: (**a**) three sets of sine wave signals; (**b**) three-sine-wave composite signal.

To validate the effectiveness of the array time-delay-based MUSIC algorithm in extracting single-frequency signals, a simulation is conducted involving three sine signals. These signals have respective amplitudes of 0.5 V, 1 V, and 0.5 V, with initial phases of −π/12, 0, and π/12. They exhibit frequencies of 3 kHz, 7 kHz, and 9 kHz, and are sampled at a 10 MHz rate. Figure 2a presents the individual sine waves, whereas Figure 2b shows the composite waveform resulting from the amalgamation of these three signals.

**Figure 3:**
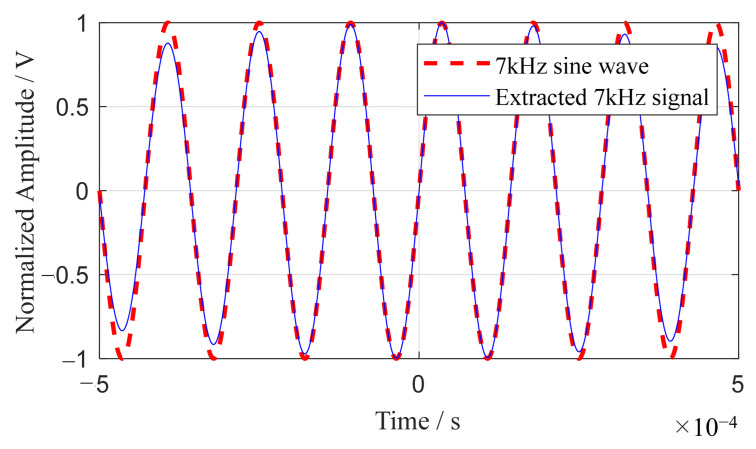
Extracted single frequency component of 7 kHz.

The array time-delay-based MUSIC algorithm’s capacity for single-frequency signal extraction is validated by its isolation of a 7 kHz frequency component. In this study, signals with a bandwidth-to-center frequency ratio of 0.1 or less are classified as effectively single-frequency. Aligning with this, the Shannon wavelet is configured to a center frequency (*f_z_*) of 1 Hz and a bandwidth (*f_k_*) of 0.1 Hz. This configuration isolates a 7 kHz component, which is then amplitude-normalized, as depicted in Figure 3. The alignment of the extracted 7 kHz signal with its original 7 kHz sinusoidal counterpart affirms the array time-delay-based MUSIC algorithm’s effectiveness in single-frequency signal extraction.

### 3.2. Comparison of Time Delay Acquisition Accuracy

The section commences with an introduction of two methods, namely, the fixed threshold method [40] and the adaptive threshold method, which are targeted at diminishing noise interference and minimizing wave packet overlap in time delay measurements. Following this, the section details experiments where array elements, uniformly spaced, are utilized to capture impact response signals. The final part of the section is an evaluation of the effectiveness of the array time-delay-based MUSIC algorithm in acquiring time delays, set in comparison with the initially mentioned fixed and adaptive threshold methods.

(1) Fixed threshold method

**Figure 4:**
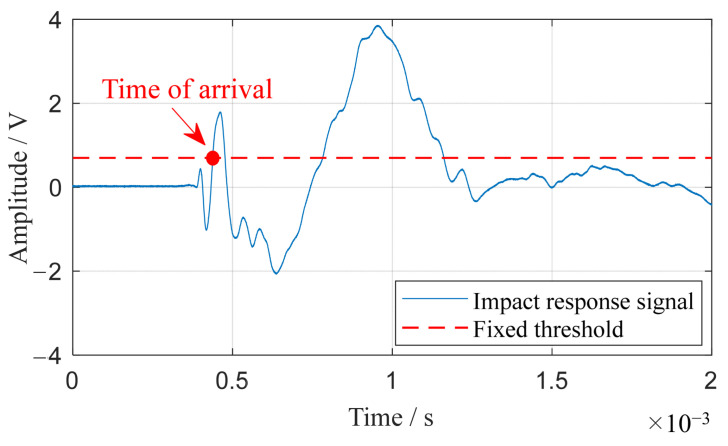
Fixed threshold method for arrival time determination.

The accurate identification of the impact moment by each sensor is key to precisely assessing time delays. In environments with high noise, traditional methods such as wave peak measurement tend to lose accuracy [41]. Moreover, techniques like wavelet analysis and cross-correlation often struggle with aliasing in guided Lamb wave packets [42]. To address these challenges, a more reliable fixed threshold method is employed for identifying the arrival of the impact signal. This method circumvents the peak method’s limitations, particularly its sensitivity to noise in small amplitude variations near the peak, as underlined in reference [41]. It also sidesteps the aliasing issue common in wave packets. By setting the threshold above the noise level, the signal’s arrival time is pinpointed with greater precision. Figure 4 visually demonstrates this technique: the impact response signal is depicted by the blue curve, while the set threshold is marked by the red dashed-line. The signal’s arrival time is denoted by a red dot, positioned at the point where the data first meet the threshold.

(2) Adaptive threshold method

Unlike the traditional threshold method, the adaptive threshold method dynamically adjusts the threshold based on the signal characteristics. In the adaptive threshold method, the first sub-peak in the wave of each signal is detected. Then, the adaptive threshold is obtained by multiplying the amplitude of the sub-peak by an adaptive coefficient. Subsequently, the time-domain signal waveform is compared to this adaptive threshold. When the signal amplitude at a certain moment *T* in the signal first reaches the adaptive threshold, it can be considered that the signal has reached the receiving sensor. Therefore, the time *T* corresponding to this amplitude is the moment at which the signal arrives.

Using the adaptive threshold method, the first step involves calculating the ratio *R_i_* between the peak amplitude *V_b_* before the sub-peak and the sub-peak amplitude *V_p_* for each sensor signal. The calculation formula for the *i*th sensor is as follows:

(12) $$R_{i}=V_{b}/V_{p},$$

To ensure that the coefficient is configured to satisfy the condition where the threshold of the adaptive threshold method is greater than the peak amplitude before the sub-peak, the coefficient *C* for the adaptive threshold method is set to be greater than all *R_i_* values and less than 1.

Once the adaptive coefficient *C* is determined, the formula for calculating the adaptive threshold (*ATH*) is as follows:

(13) $$ATH=C\times V_{p},$$

For the guided Lamb wave signal *s*(*t*) with a sampling rate of *f_s_* and a sampling length of *m*, and given the *ATH*, the formula for calculating the moment of signal arrival is as follows:

(14) $$T=\arg\underset{t}{\min}\{s(t-1)<ATH\leq s(t)\},\;t=\frac{1}{f_{s}},\frac{2}{f_{s}},\frac{3}{f_{s}},\cdots ,\frac{m}{f_{s}},$$

**Figure 5:**
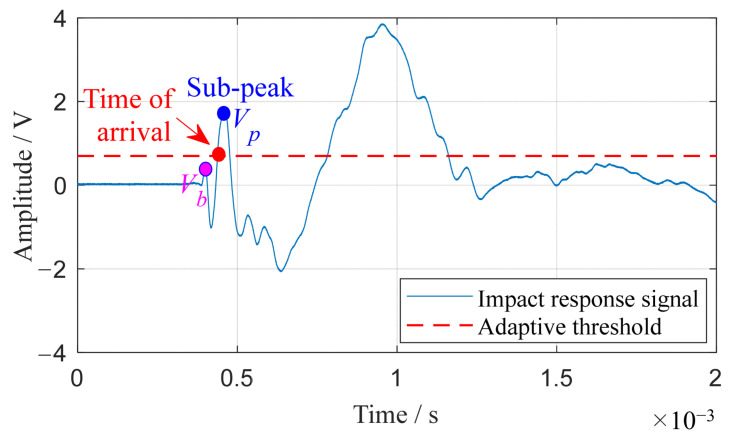
Adaptive threshold method for arrival time determination.

Figure 5 illustrates a typical example demonstrating the determination of signal arrival time using the adaptive threshold method. Initially, the sub-peak of the signal is identified. Subsequently, the adaptive threshold is obtained by multiplying the sub-peak amplitude by *C*, as depicted by the red dashed-line in Figure 5. Finally, the wave arrival time is represented by the red dot in Figure 5.

(3) Single-frequency signal extraction method

**Figure 6:**
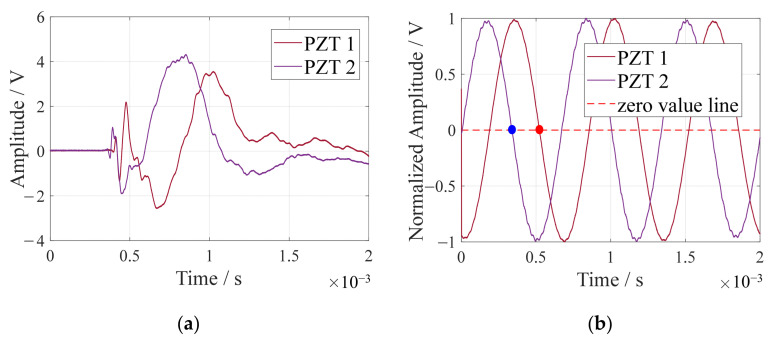
Single-frequency signal extraction for time delay determination: (**a**) impact response signals; (**b**) extracted single frequency signals.

Based on the array time-delay-based MUSIC algorithm for time delay acquisition, the signal’s component with the highest amplitude at a single frequency is first isolated. Time delay measurement involves calculating the time difference between consecutive zero crossings in this specific single-frequency signal. Figure 6a displays the impact response signals from two array elements, PZT 1 and PZT 2. In Figure 6b, the derived single-frequency signals, obtained using the Shannon wavelet method, are presented. The time delay between PZT 1 and PZT 2 is marked by the interval between the solid blue and red dots in Figure 6b.

(4) Evaluation of different time delay acquisition methods

An experiment is performed to assess the precision of the proposed method in determining array time delays. In this experiment, a linear array of elements uniformly spaced receive impact response signals from the same direction at equal intervals. The method’s effectiveness is then compared with fixed threshold and adaptive threshold techniques to highlight its accuracy.

**Figure 7:**
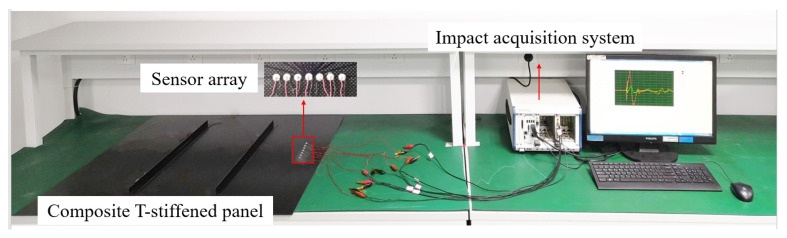
Experimental setup and sensor array configuration.

The experiment focuses on a composite material T-stiffened panel depicted in Figure 7. This panel, measuring 900 mm by 900 mm with a thickness of 3 mm, incorporates two T-stiffeners along its length for added rigidity. The stiffeners are spaced 260 mm apart, and each is 125 mm from the panel’s top and bottom edges. Its surfaces are reinforced with plain weave carbon fiber cloth, enhancing durability and smoothness. The panel’s symmetric laminate structure follows a [45/0/-45/90/0/45/0/-45/0] _S_ layup sequence, indicating multiple ply orientations. The areal weight of the fabric is 150 g/m^2^, and the matrix polymer is epoxy resin. Seven piezoelectric sensors, each 8 mm in diameter and 0.48 mm thick, are affixed to form a one-dimensional, evenly spaced linear array over a length of 72 mm, with 12 mm gaps between sensors. These sensors, named sequentially from PZT-3 to PZT 3, are aligned from left to right.

**Figure 8:**
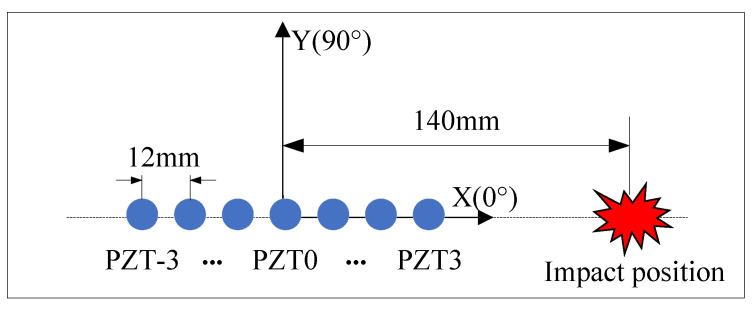
Experimental layout for time delay measurement.

To evaluate the performance of three time delay acquisition methods, a controlled variable method is used to eliminate the effects of anisotropy in composite material structures. An evaluation experiment is conducted where seven elements of a linear array receive impact response signals at equal intervals from the same direction. The layout of the experiment, as illustrated in Figure 8, shows that the distance between the impact location and PZT 0 is 140 mm. An impact force hammer is used in the experiment to simulate low-velocity external impacts.

**Figure 9:**
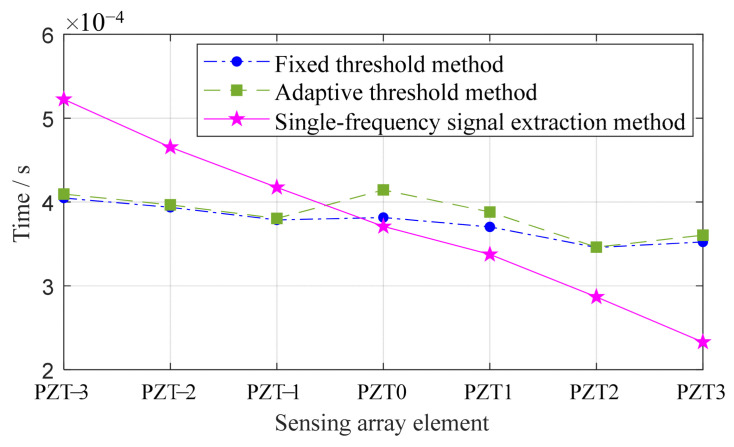
Arrival times of the three methods.

The arrival times of impact signals received by each array element are ascertainable using three methods: the fixed threshold method, the adaptive threshold method, and the single-frequency signal extraction method. When employing the single-frequency signal extraction method, the extracted signal frequency is 5.5 kHz. These methods’ effectiveness in time delay acquisition is showcased in Figure 9. Ideally, there should be a sequential delay in the impact signal’s arrival time at each element, moving from PZT 3 to PZT -3. The analysis reveals that the single-frequency signal extraction method demonstrates greater reliability compared to the other two. Additionally, a consistent upward trend in arrival times is noted as the distance from the impact source to each array element increases.

## 4. Impact Localization on a Composite Structure

The theoretical analysis in Section 2 highlights velocity as a key input for the MUSIC algorithm. Due to the anisotropy in composite materials, the velocity of guided Lamb wave propagation in different directions of the structure can vary significantly. Prior to any impact, the exact velocity for the algorithm is indeterminable. Consequently, it is essential to pre-measure velocities in various directions on an intact structure. Post-impact, an initial estimation of the impact angle is derived from the average velocity across these directions, aiding in preliminary impact site localization. Subsequently, the closest measured guided Lamb wave velocity to the initially estimated angle is selected to represent the impact direction. This selection facilitates recalculating the array steering vector, leading to the precise determination of the angle and distance at the spatial spectrum’s spectral peak, indicative of the impact source’s exact location. Following, the paper details the methodology for measuring velocity in different directions within the structure, and discusses the impact localization results achieved using the MUSIC algorithm with array time delays.

### 4.1. Single-Frequency Signal Velocity Measurement

This method measures single-frequency signal velocity in structures using piezoelectric sensor pairs. First, the sensors are positioned on the structure, and an impact is applied at the angle created by these sensors. The sensors then capture the impact signals. Single-frequency signals are isolated using the Shannon wavelet transform, and the time difference between them is determined. The velocity of these single-frequency guided Lamb waves is calculated by incorporating the distance between the sensors. A detailed explanation of this single-frequency signal velocity measurement technique, based on single-frequency signal extraction, follows.

**Figure 10:**
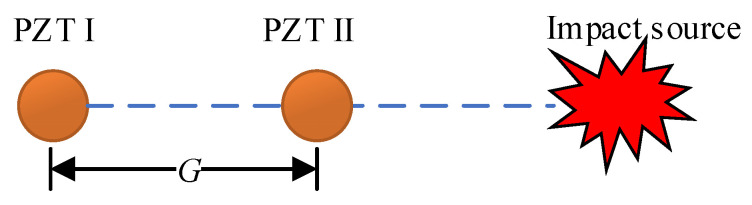
Configuration for measuring the velocity of single-frequency signals.

Initially, as illustrated in Figure 10, piezoelectric sensor pairs PZT I and PZT II are positioned on the structure for the purpose of measuring the velocity of guided Lamb wave single-frequency signals. It is essential to maintain the phase difference of signals from PZT I and PZT II within the wavelength range of the guided Lamb wave at their corresponding frequency. This implies that the distance *G* between the sensor pair should be less than the wavelength of the guided Lamb wave at that frequency.

After positioning the sensors, an impact is applied along the line joining them. The response signals are then captured by the impact acquisition system. This system gathers the impact response signals from both sensors, after which single-frequency signals are isolated using the Shannon wavelet transform. The parameters for this transform are set with a frequency width (*f_k_*) of 0.1 Hz and a central frequency (*f_z_*) of 1 Hz, achieving a frequency width to center frequency ratio of 0.1. The process involves extracting two single-frequency signals of the target frequency within the same cycle. The phase difference is measured between adjacent zero-crossing points of these signals, providing the time delay (Δt) between the signals received by PZT I and PZT II. Finally, using Equation (15) and the distance (G) between the sensors, the velocity (v) of the guided Lamb wave’s single-frequency signal at the specific frequency can be accurately determined.

(15) v=G/Δt

**Figure 11:**
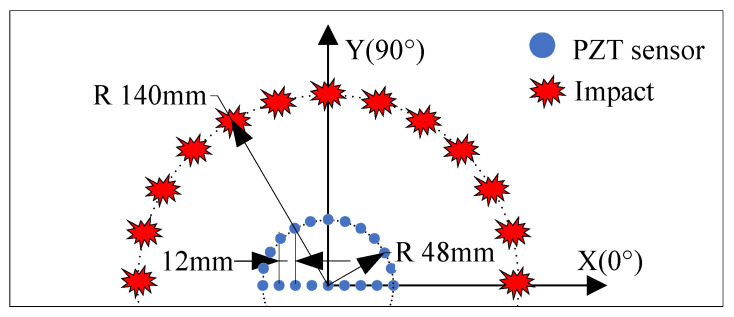
Measuring the velocities of single-frequency signals in various directions.

To measure the velocities of single-frequency signals in various directions within the structure, as depicted in Figure 11, an additional 13 piezoelectric sensors are positioned on the structure. These sensors are arranged at 15-degree angular intervals, forming a semi-circle 48 mm away from the central reference element, PZT 0. To determine the single-frequency signal velocity, impacts are applied at angles corresponding to both the additional sensors and PZT 0. The response signals are then recorded by the two sensors. The single-frequency extraction method is used to isolate the required single-frequency components from these impact response signals. The time delays between these signals are measured, and by correlating these delays with the known distance between the sensors, the velocity of the guided Lamb wave’s single-frequency signals is calculated.

**Figure 12:**
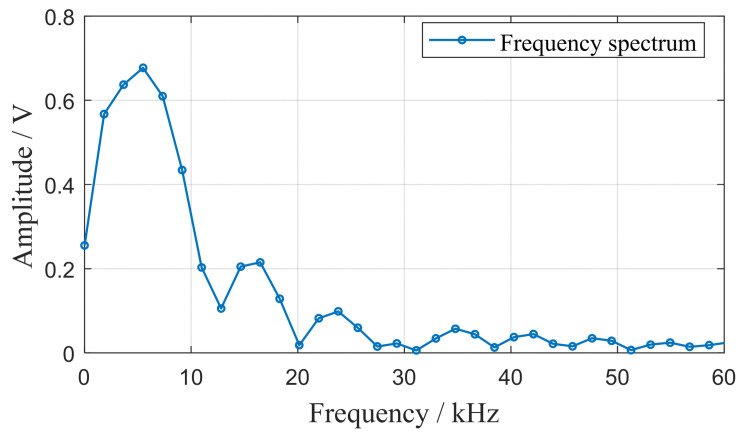
Reference array element spectrogram.

Consider the example of applying an impact at the position (140 mm, 90°) on the structure to demonstrate the process. Initially, a Fourier transform is conducted to analyze the spectrum of the impact signal generated at this location. Figure 12 presents the spectrum of the impact response signal from the reference array element PZT 0. This analysis reveals that the broadband impact response signal is intricate, showing that the main energy distribution of the impact predominantly occurs within the 10 kHz range.

**Figure 13:**
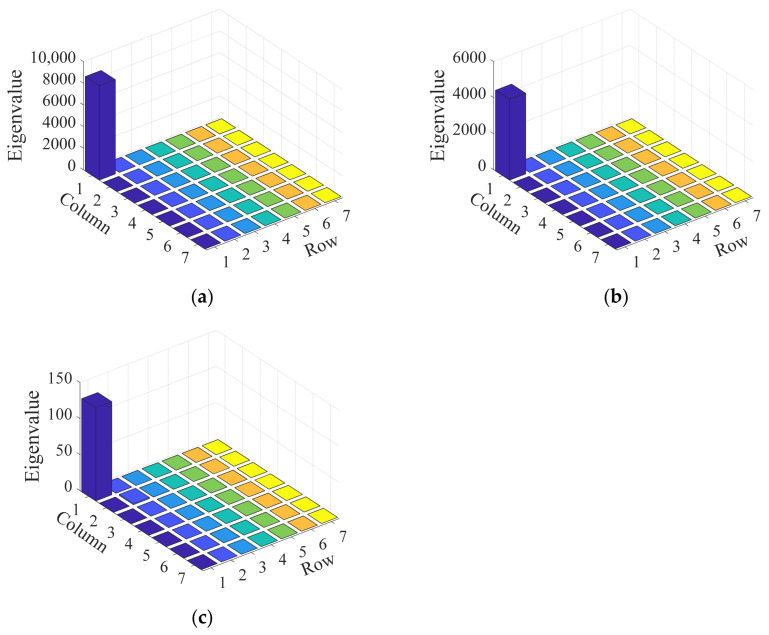
Distribution of covariance matrix eigenvalues across various extraction frequencies: (**a**) 5.5 kHz; (**b**) 10 kHz; (**c**) 30 kHz.

**Figure 14:**
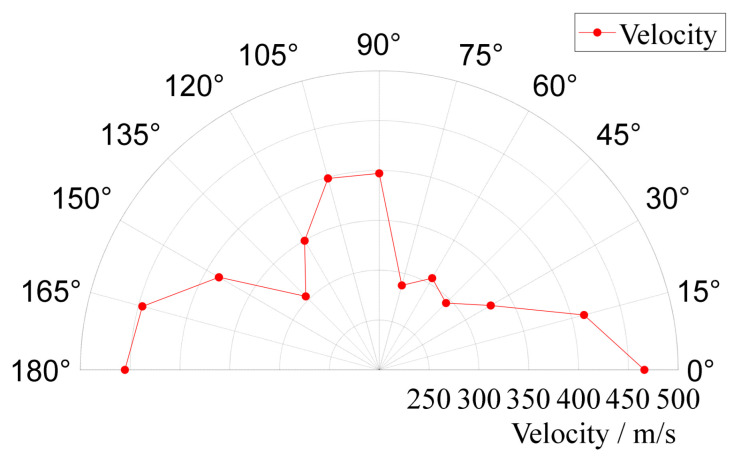
Velocities of single-frequency signals in various directions.

The eigenvector corresponding to the covariance matrix’s maximum eigenvalue is recognized as indicative of the array signal’s propagation direction. The eigenvalues of the covariance matrix are indicative of the magnitude of the array signal’s projection onto the associated eigenvector. A comparison of eigenvalues at different frequencies of the extracted impact response signals is conducted. In Figure 13, the distribution of eigenvalues at extraction frequencies of 5.5 kHz, 10 kHz, and 30 kHz is presented. Significant variations in eigenvalues are observed across signals at various extraction frequencies, with the largest disparity being an order of magnitude difference between the maximum eigenvalues at 10 kHz and 30 kHz. It is suggested that the larger the amplitude at a given frequency, the greater the corresponding eigenvalue. As a result, the single-frequency signal at 5.5 kHz, characterized by a higher energy content, is selected by the algorithm to enhance the disparity in the magnitudes of eigenvalues. The Shannon wavelet function, with a mother wavelet center frequency of 1 Hz and a frequency width of 0.1 Hz, is employed. The velocities of single-frequency signals measured in different directions within the structure are illustrated in Figure 14.

### 4.2. Impact Localization Results

**Figure 15:**
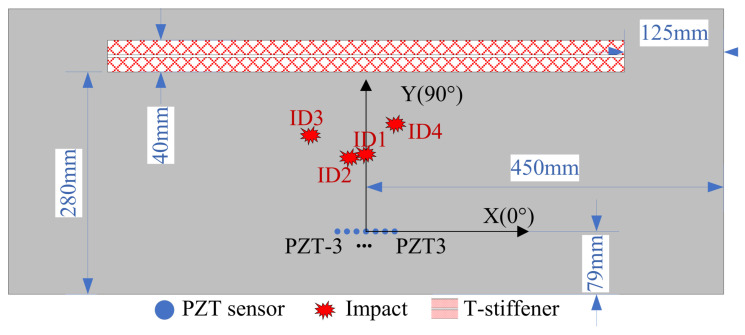
Schematic diagram of the impact positioning experiment.

**Figure 16:**
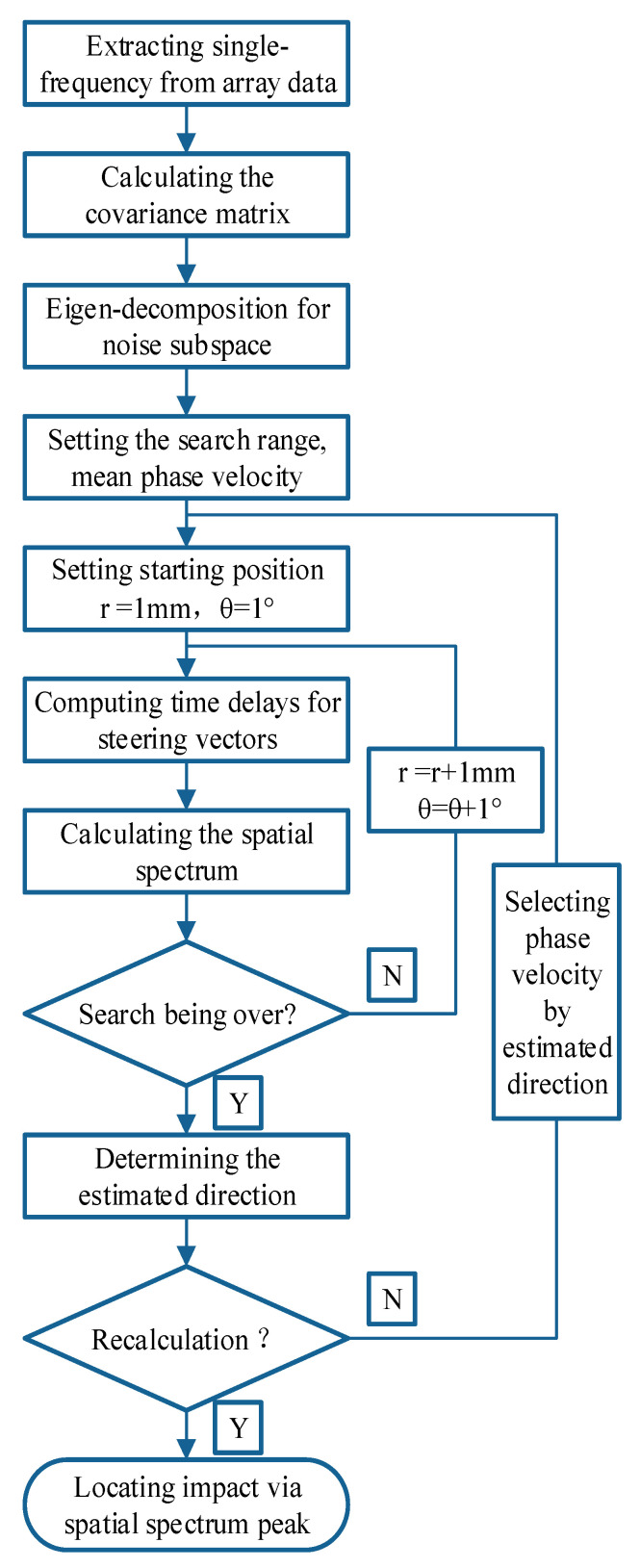
Flowchart of the developed MUSIC algorithm.

**Figure 17:**
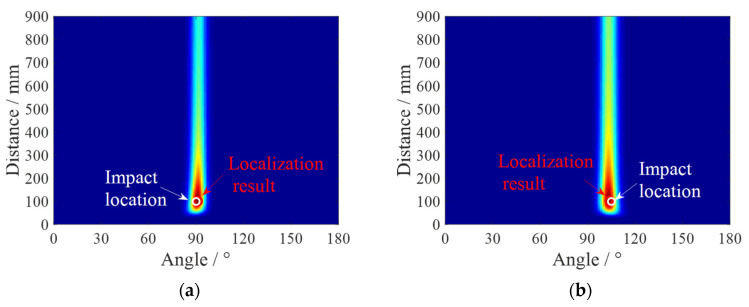

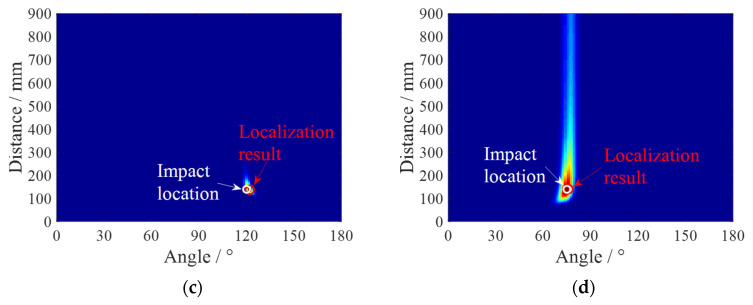
Spatial spectrums of impacts: (**a**) ID1; (**b**) ID2; (**c**) ID3; (**d**) ID4.

In the impact localization experiment conducted on a composite material T-shaped reinforced panel, four impact positions are selected as validation instances at different distances and angles. As illustrated in Figure 15, the four impact locations are situated between the reinforcement ribs and the sensor array. The uniform linear sensor array is located 79 mm from the bottom edge of the composite material T-shaped reinforced panel. Additionally, the reference sensor element, PZT0, is equidistant from the left and right boundaries of the composite material T-shaped reinforced panel, at 450 mm from each side. The coordinates of the impact points are ID1 (90°, 100 mm), ID2 (105°, 100 mm), ID3 (120°, 140 mm), and ID4 (75°, 140 mm). The array time-delay-based MUSIC algorithm is applied. Figure 16 presents the flowchart of the developed MUSIC algorithm. The method initiates by employing the average phase velocity to preliminarily estimate the angle of impact, thereby approximating the impact’s angular position. Subsequently, leveraging the estimated angle of impact, it selects the guided Lamb wave phase velocity corresponding to that angle to precisely localize the impact. This approach effectively addresses the anisotropy in the composite structure, thereby enhancing the positioning accuracy of the MUSIC algorithm. By setting the search region with a step size of 1 mm from 0 mm to 900 mm in distance and a step size of 1° to 180° in angle, spatial spectra for the search region are obtained. Figure 17 illustrates the spatial spectrum estimation for the four impact sources, where the horizontal axis corresponds to the angle of the impact source, and the vertical axis corresponds to the distance of the impact source. In the figure, red circles indicate the estimated impact positions by the proposed algorithm (localization results), and white circles mark the actual impact locations. Table 1 lists the localization results and error statistics for these impacts. The maximum localization error is found to be 2 mm in distance and 2° in angle, demonstrating the high accuracy of the array time-delay-based MUSIC algorithm in impact localization.

## 5. Conclusions

This paper proposes an innovative time-delay-based MUSIC algorithm for impact imaging, utilizing a guided Lamb wave array. The algorithm, by extracting high-energy single-frequency components from impact response signals, not only improves the accuracy of time delay measurements across array elements, but also significantly enhances noise resistance. Additionally, the method involves calculating the average velocity of single-frequency components in various directions, aiding in the initial estimation of the impact angle. This estimation is vital for selecting the correct single-frequency velocity, enabling precise localization of the impact position and effectively addressing the anisotropic challenges in composite material structures. The experimental evaluation, conducted on reinforced composite structures using an array of equidistantly spaced elements, demonstrates the algorithm’s effectiveness in accurately determining array time delays. The impact localization tests further underscore the high precision of the algorithm in pinpointing the locations of impacts.

In practical engineering applications, the propagation of guided Lamb waves is influenced by external environmental factors, which may further diminish the localization accuracy of the MUSIC algorithm. Future work needs to investigate the impact of these environmental factors on the MUSIC algorithm, consider the MUSIC impact localization method under time-varying conditions, and conduct corresponding experimental validations.

## Figures and Tables

**Table 1:** Localization results of the impacts.

Impact	Locations	Array Time-Delay-Based MUSIC Algorithm			
		Localization Results		Error	
		Angle	Distance	Angle	Distance
ID1	(90°, 100 mm)	91°	101 mm	1°	1 mm
ID2	(105°, 100 mm)	104°	102 mm	1°	2 mm
ID3	(120°, 140 mm)	122°	138 mm	2°	2 mm
ID4	(75°, 140 mm)	76°	141 mm	1°	1 mm

## Data Availability

No new data were created or analyzed in this study. Data sharing is not applicable to this article.
